# Towards Sustainable Recycling of Epoxy-Based Polymers: Approaches and Challenges of Epoxy Biodegradation

**DOI:** 10.3390/polym15122653

**Published:** 2023-06-12

**Authors:** Leon Klose, Neele Meyer-Heydecke, Sasipa Wongwattanarat, Jennifer Chow, Pablo Pérez García, Camille Carré, Wolfgang Streit, Garabed Antranikian, Ana Malvis Romero, Andreas Liese

**Affiliations:** 1 Institute of Technical Biocatalysis, Hamburg University of Technology, 21073 Hamburg, Germany; 2 Department of Microbiology and Biotechnology, University of Hamburg, 22609 Hamburg, Germany; 3 Airbus Defence and Space GmbH, Central Research and Technology, 81663 Munich, Germany

**Keywords:** epoxy, carbon-fiber-reinforced polymers, biodegradation, degradation analytics, plastic recycling

## Abstract

Epoxy resins are highly valued for their remarkable mechanical and chemical properties and are extensively used in various applications such as coatings, adhesives, and fiber-reinforced composites in lightweight construction. Composites are especially important for the development and implementation of sustainable technologies such as wind power, energy-efficient aircrafts, and electric cars. Despite their advantages, their non-biodegradability raises challenges for the recycling of polymer and composites in particular. Conventional methods employed for epoxy recycling are characterized by their high energy consumption and the utilization of toxic chemicals, rendering them rather unsustainable. Recent progress has been made in the field of plastic biodegradation, which is considered more sustainable than energy-intensive mechanical or thermal recycling methods. However, the current successful approaches in plastic biodegradation are predominantly focused on polyester-based polymers, leaving more recalcitrant plastics underrepresented in this area of research. Epoxy polymers, characterized by their strong cross-linking and predominantly ether-based backbone, exhibit a highly rigid and durable structure, placing them within this category. Therefore, the objective of this review paper is to examine the various approaches that have been employed for the biodegradation of epoxy so far. Additionally, the paper sheds light on the analytical techniques utilized in the development of these recycling methods. Moreover, the review addresses the challenges and opportunities entailed in epoxy recycling through bio-based approaches.

## 1. Introduction

Epoxy polymers are widely used in various industries, e.g., as coatings, adhesives, and for lightweight construction due to their unique properties such as high strength, chemical resistance, and adhesion to various surfaces [1,2]. Therefore, one of the most prominent applications is their use as matrix material in fiber-reinforced composites, which are heavily employed in the aerospace sector [3]. However, the disposal of epoxy polymers and composites thereof has become a significant concern due to their recalcitrant nature and the adverse environmental effects caused by traditional recycling methods [4].

**Figure 1:**
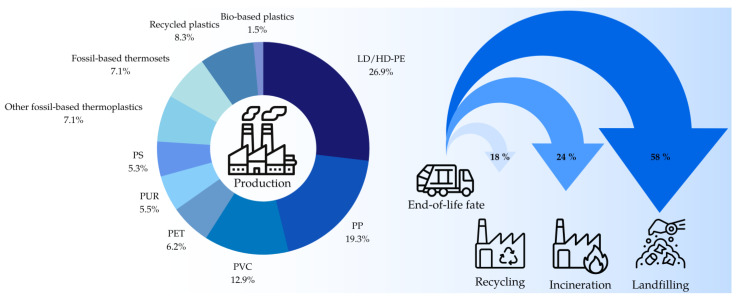
Production volume (status 2021) and end-of-life fate of plastics (status 2017) [7,11]; LD/HD-PE—Low-density/High-density polyethylene, PP—Polypropylene, PVC—Polyvinyl chloride, PET—Polyethylene terephthalate, PUR—Polyurethane, PS—Polystyrene.

In this context, the overall production of plastic waste is projected to double within the next 20 years, with only 18% currently being recycled, leading to the deposition of around 12,000 Mt of plastic waste in landfills and the environment by 2050 (see Figure 1) [5,6,7]. Even though epoxy polymers only accounted for a rather small share of the global plastic production volume of around 7.1% in 2021, the demand for fiber-reinforced polymers is steadily increasing [8]. This is closely linked to emerging sustainable technologies such as off- and on-shore wind power plants or electric vehicles that rely on lightweight construction materials for increased efficiency [9]. To cover the resource demand of the composite industry and to reduce the environmental impact of accumulated epoxy waste, there is an urgent need for the development of sustainable recycling methods.

Conventional recycling methods for epoxy polymers, such as solvolysis, pyrolysis, or nitric acid treatment, involve harsh chemicals and the application of high temperatures and pressures [10]. Not only does this lead to the emission of large quantities of CO_2_ and other pollutants, but it also generally results in a reduction of the material properties. Particularly with regard to composites, the fibers often suffer from the extreme reaction conditions, resulting in a reduction in their quality [4].

As a result, plastic-degrading biocatalysts have attracted growing attention due to their advantageous properties such as milder process conditions, high substrate specificity, and overall reduced environmental impact [12,13,14]. Enzymatic PET (polyethylene terephthalate) depolymerization is one example of the significant progress that has been made within the last decade in the field of plastic degradation [15,16]. However, the development of biodegradation approaches for recalcitrant plastics such as epoxy polymers has been less comprehensive and is still limited to a few studies undertaking this challenge [17,18]. In this regard, the exploitation of biodiversity resources, especially in combination with metagenome mining and environmental screenings, represents a promising approach towards the development of novel biocatalysts for plastic degradation [19,20,21,22,23,24]. This approach has been successful in identifying enzymes that can degrade plastics such as PET and can potentially be used to develop biocatalysts for the degradation and recycling of epoxy polymers [25].

Thus, this review aims to provide an overview of the approaches taken towards the development of biochemical degradation methods for epoxy and epoxy-based fiber-reinforced composites. As the development of bio-based recycling technologies also requires comprehensive analysis of the underlying processes, an overview of available analytical approaches is given.

## 2. Epoxy Polymers

### 2.1. Production

Epoxy resins belong to the class of thermoset polymers and possess a strong dimensional stability which is based on the cross-linking of the polymer [26]. The preparation of an epoxy resin involves the reaction between an epoxide monomer and a curing agent. The epoxide monomer usually contains two or more oxirane (epoxide) groups that can react with a variety of curing agents, such as aliphatic and aromatic amines, amides, phenols, and acids, to form a cross-linked polymer network. The curing agents can also contain multiple reactive sites that participate further in the cross-linking reaction. The choice of the curing agent and the reaction conditions can significantly affect the properties of the resulting epoxy resin [27]. Epoxy resins are available with various backbones and can be tailored for specialized applications by different chemical properties and degrees of cross-linking [28].

**Figure 2:**
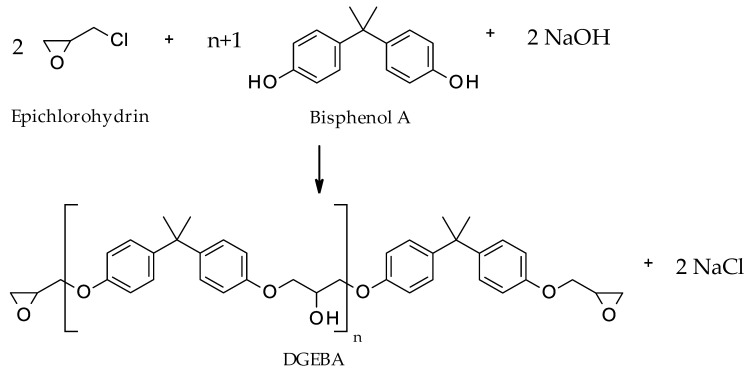
Bisphenol A based synthesis of bisphenol A diglycidyl ether (adapted from [30]).

The majority (>75%) of epoxy resins are based on bisphenol A (BPA) and its derivatives as a reactive subunit. An overview of the underlying polymerization reaction is given in Figure 2. In an initial addition reaction, two epichlorohydrin molecules were attached to BPA, after which two new epoxide residues were formed by a condensation reaction. The chlorine atom was removed as NaCl, while the residual hydrogen atom forms a water molecule in the basic environment [29]. The resulting bisphenol A diglycidyl ether (BADGE or DGEBA) is referred to liquid epoxy resin and represents the monomeric unit of the polymer. The polymerization is initiated by further reaction with BPA, leading to the formation of a polyether backbone. The increase in molecular weight of the linear polymer chains causes an increase in viscosity, which gradually leads to the solidification of the resin [30].

**Figure 3:**
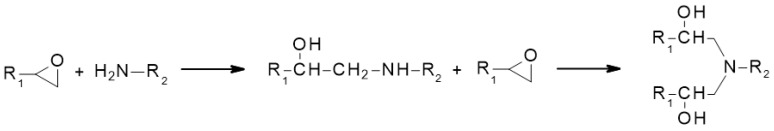
Cross-linking of epoxy components with an arbitrary amine hardener (adapted from [32]).

To receive a strong and completely solid product, the use of hardeners (also referred to as cross-linking or curing agents) is mandatory [31]. These are divided into two categories, declaring them either as catalytic or co-reactive. Catalytic hardeners initiate and accelerate the homo-polymerization of the epoxy resin and are therefore not present within the polymer after the curing process. Examples for catalytic hardeners are Lewis acids and bases such as boron trihalides and tertiary amines. If a stronger degree of crosslinking is required, co-reactive curing agents with active hydrogen atoms such as primary and secondary amines, thiols, or carboxylic acids can be employed [32,33]. These react as co-monomers in the polymerization, leading to a three-dimensional reinforcement of the typically linear epoxy chains, as depicted in Figure 3. The curing process usually involves the application of a heating gradient that initiates bond formation among the subunits [30,34].

### 2.2. Applications

Epoxy polymers are widely used in various industries due to their exceptional mechanical and thermal properties. They are commonly used as matrix materials in composites, which are extensively applied in the aerospace and automotive industries to produce lightweight, high-strength parts [35]. Epoxy resins are also popular as adhesives, sealants, and coatings due to their excellent adhesive properties and chemical resistance [36]. They are used to bond a variety of materials, making them essential in many industrial applications, particularly in the construction industry, where they are used to repair and strengthen concrete parts or protect steel structures from corrosion and wear [37].

In the electronics industry, epoxy polymers are used as encapsulants and potting materials for electronic components. They are particularly suitable for use in high-frequency and high-voltage applications, as they have a high dielectric strength and low dielectric constant [38]. Therefore, they are widely used in the manufacturing of printed circuit boards (PCBs), where they serve as adhesives to bond various components and coatings to protect the PCB from moisture, dust and other environmental factors. Additionally, their high thermal resistance makes them ideal for use in LED encapsulation, which is increasingly replacing traditional encapsulation methods [39].

**Figure 4:**
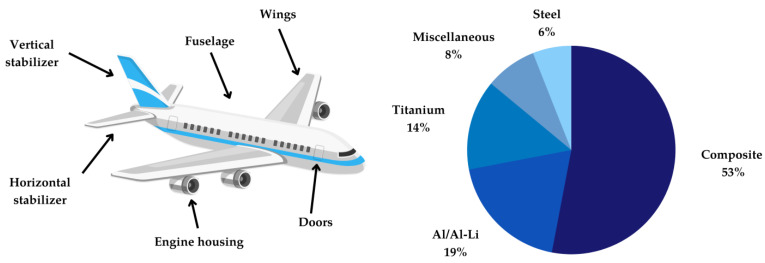
Overview of the parts manufactured from composites and percentage composition of the construction materials of the Airbus A350XWB (adapted from [43]).

One of the broadest application fields of epoxy polymers are as building and construction material in fiber-reinforced composites [29]. As such, they are applied in the manufacturing of airplanes, sports equipment such as skis, snowboards, and surfboards, wind turbine blades, electric vehicles, and more. The high strength and stiffness of these materials make them ideal for use in these applications, where weight reduction and durability are crucial. Various types of fibers such as carbon, glass, aramid, and natural fibers can be used in fiber-reinforced polymers [40]. However, carbon-fibers have the benefit of having a high strength and stiffness, low weight, and excellent fatigue resistance, making them an ideal choice for reinforcing composites in high-performance applications. The Airbus A350XWB, for example, is made of 53% carbon-fiber-reinforced polymers (CFRP), which are predominantly used in fuselage and wing components (see Figure 4) [41]. This makes it possible to achieve significant weight savings of up to 50% for certain components in comparison to the use of aluminum, which is reflected in the efficiency of modern aircrafts [3].

To achieve superior material properties in terms of stiffness, tensile strength, and Young’s modulus, among others, the carbon-fibers are embedded into the polymeric epoxy matrix. Nowadays, these fibers are predominantly produced from polyacrylonitrile, a long-stranded polymer with a carbon backbone. The thin polymeric fibers are carbonized at temperatures of 1000–1800 °C in an inert atmosphere to enrich the carbon content and the degree of crystallinity within the strands [42].

By different physicochemical methods, the surface of the fibers is etched to increase the roughness and promote the interaction with the polymeric matrix in later production steps [44]. Afterwards, the fibers are woven into fabric sheets, which allows for an even integration into the epoxy resin. The composite can be manufactured using various methods, including pre-preg and liquid composite molding (LCM). Pre-preg involves impregnating the carbon-fibers with a resin before curing and is commonly used in the aerospace industry for producing high-quality and high-strength parts [29]. In LCM, the resin was injected into a mold containing dry carbon-fibers, which was then cured under heat and pressure. Resin transfer molding (RTM), a type of LCM, involves injecting resin into a closed mold. The resin flows through a preform of dry fibers, thus impregnating them [45]. Vacuum bagging is another cost-effective LCM method that involves placing the carbon-fibers and resin in a mold and removing air with a vacuum bag, which results in a good surface finish and low void content [46].

In Table 1, the mechanical properties of CFRPs are compared to those of plain epoxy and steel. Both the tensile strength and Young´s modulus of certain CFRPs can be extensively compared to steel and pure epoxy. This is achieved by a distribution of the load throughout the composite, which is reinforced by a strong bonding between the fibers and the matrix [47]. Along with the up to three-fold lower density of CFRPs in comparison to steel, the elongation of the material under mechanical stress can be as low as 0.5%, while the parameter of steel can reach up to 12%. Even though CFRPs are superior to metallic materials in many respects, these materials have their weak points, especially in terms of low compressive and radial strength, which are caused by the anisotropic nature of the composite [48].

The increased use of CFRPs and fiber-reinforced epoxy-based composites therefore pose greater recycling challenges, as the matrix and fibers must be separated. However, since conventionally used fibers for reinforcement are of non-renewable origin, the recycling of the composites has an even higher resource potential, since the fibers and the epoxy matrix components can be recovered [50].

### 2.3. Conventional Epoxy Recycling Approaches

Plastic recycling refers to the recovery and reprocessing of waste plastics into new products. These do not necessarily need to resemble the initial state. Recycling plays a critical role in the context of a circular economy by reducing waste and conserving resources. In a circular economy, waste is minimized, and products are designed to be reused or recycled at the end of their life cycle. By recycling materials, valuable resources are kept in use and the need for extracting new resources is reduced, promoting sustainability and reducing the environmental impact of production [50].

**Figure 5:**
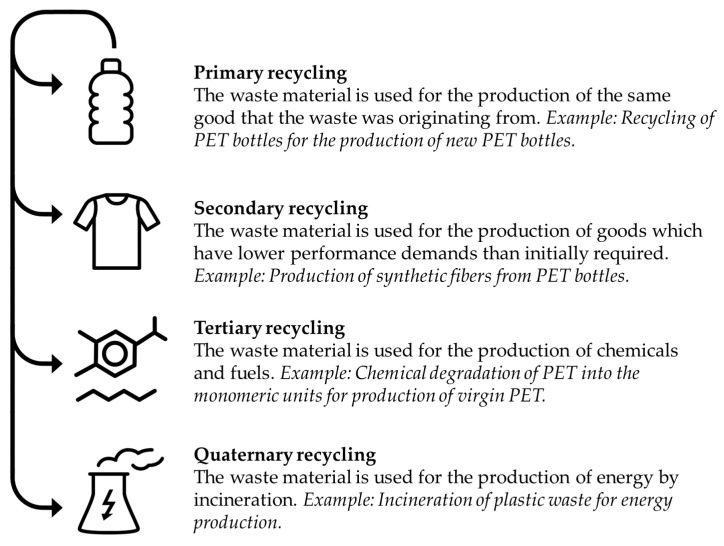
Comparison of the four recycling categories (adapted from [51]).

Current strategies for the recycling of plastics can be divided into four categories according to Merrington [51] (see Figure 5).

Primary recycling is, in most cases, the desired method to re-utilize plastic waste, since typically only one specific type of polyolefin needs to be processed. The recycling, however, becomes more challenging when other plastics or additives such as pigments, plasticizers, or fibers are present in the materials [52]. Additionally, primary and secondary recycling comprises mostly of mechanical and thermal processing steps which significantly reduce the mechanical properties of the plastics, leading to a product of lower quality. It is, therefore, also only applicable to thermoplasts such as polyethylene (PE), polypropylene (PP), polyvinyl chloride (PVC), polystyrene (PS), and PET. As these materials are composed of chain-like molecules interacting with each other through weak van der Waals forces, they can be molten and re-extruded. Thermoset polymers, such as epoxy, phenol formaldehyde, and benzoxazine resins, are accordingly excluded from these recycling steps due to their cross-linked macromolecular nature [53]. Since quaternary recycling, which describes the incineration of the waste for energy production, is hardly an option in the sense of a circular polymer economy, only a few recycling options remain for thermoset polymers.

**Figure 6:**
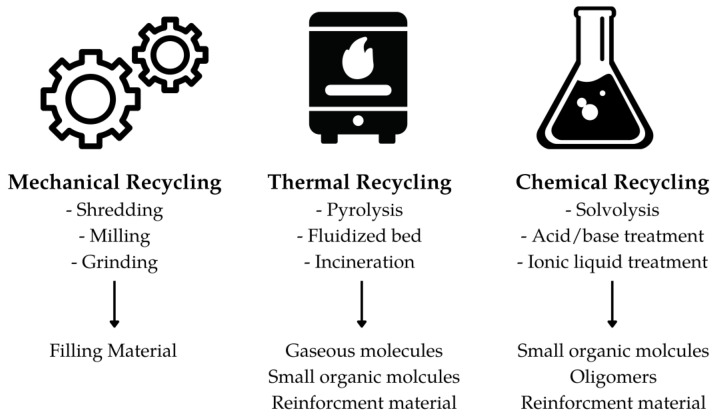
Conventional epoxy/epoxy composite recycling processes.

Conventional recycling methods (see Figure 6) for epoxy resins include chemical, mechanical, and thermal treatment. Chemical treatment involves breaking down the epoxy polymer into smaller molecules through incubation with strong catalysts such as NaOH, peracetic acids, nitric acid, ionic liquids, or metal catalysts [54,55,56,57]. However, the high energy consumption and hazardous waste generated during the process limit its practicality and applicability. Mechanically recycled waste material is ground into small particles and can be reused to produce low-performance products as filler material. Although it is relatively simple and cost-effective, the resulting recycled material properties are significantly lower than the original material, limiting its application to low-value products [58].

Thermal recycling involves burning the waste material to produce energy, also known as waste-to-energy (WtE) conversion. However, this process also results in the emission of pollutants, which can have severe environmental impacts [59]. Another thermal recycling method is pyrolysis, which involves heating the waste material to high temperatures in the absence of oxygen, causing it to break down into gas and char [60]. While this method is less polluting than traditional WtE methods, it still requires a high energy input and produces hazardous waste. These conventional recycling methods have several shortcomings, including the high energy consumption and environmental impact [61]. Therefore, research in the field of biochemical recycling of plastics has intensified to develop more sustainable methods [62,63].

## 3. Biocatalytical Recycling Approaches

### 3.1. Excursus: Thermoplast Biodegradation

Based on their rapid adaption to biotic and abiotic factors, microorganisms possess the ability to degrade and utilize a plethora of organic materials. Due to their growing abundance in the environment, even materials of anthropogenic origin, e.g., synthetic polymers, can be susceptible to biodegradation. So far, a variety of bacteria, fungi, and algae have been reported to be able to degrade and metabolize synthetic polymers [64]. However, the majority of these organisms act mainly on polymers with a heteroatomic backbone, which can offer better conditions for enzymatic attacks due to their—in some, but not all cases—hydrolyzable ester backbone [65].

Table 2 gives an overview of the most common synthetic polymers and their categorization as polymer with a hetero- or homoatomic backbone. Materials such as PE, PP, PVC, and PS belong to the latter and are considered to have a higher recalcitrance to (bio-) chemical depolymerization as a result of their homoatomic C-C backbone. Typical examples of heteroatomic backbone polymers are PET, polyurethane (PUR), polylactic acid (PLA), or polyamide (PA), which consist of a backbone containing carbon, oxygen, and/or nitrogen, and are, in most cases, susceptible to hydrolysis. However, even though the epoxy polymer also has a heteroatomic backbone, the lack of ester or amide bonds increases the resistance to hydrolysis and the high degree of cross-linking [66]. Among polymers with a heteroatomic backbone, biocatalytical PET degradation has been predominantly studied. The most prominent phylum known to be capable of degrading PET is *Actinobacteria*. This includes the genera *Thermobifida* and *Thermomonospora*, which are thermophilic organisms whose proteins exhibit an increased thermal stability. The enzymes identified to be involved in the PET degradation are serine hydrolases containing a catalytic triad in their active site and harbor additional disulfide bonds that confer the thermostability to the proteins [67]. A prime example of PET depolymerization was elucidated for the bacterium *Ideonella sakaiensis*, which was isolated by Yoshida et al. [68] in an environmental sample from a PET recycling facility. It harbors the enzyme MHETase, which is capable of hydrolyzing internalized mono(2-hydroxyethyl) terephthalic acid (MHET), the PET sub-units produced by the extracellular enzyme PETase, into terephthalic acid (TPA) and ethylene glycol (EG). Thereby, a complete metabolization of the polymer and utilization of the monomers as carbon and energy source is possible.

Further enzymatic potential for the biodegradation of PET was identified in different fungal species. Cutinases, which are naturally used for the scission of cutin (protecting polyester layer on plant leaves), have been found to act on PET. Carniel et al. [69] used a cutinase (HiC) from *Humicola insolens* in combination with lipase B (CALB) from *Candida antarctica* and revealed a synergistic effect of both esterases, which led to an increased PET depolymerization in comparison to the sole use of HiC.

One of the most successful approaches in the depolymerization of PET was published by Tournier et al. [16]. The authors used computer-aided engineering to optimize leaf and branch compost cutinase (LCC), which was originally described by Sulaiman et al. [70] as a PET-degrading enzyme. With an average productivity of 16.7 g∙L^−1^∙h^−1^ based on the product terephthalic acid, they achieved a degradation rate of 90% over a period of 10 h, paving the way for the commercialization of the process.

In addition to PET, ester-based polyurethane is another example for a synthetic polymer that is susceptible to hydrolytic cleavage. Different bacterial species from the genera *Bacillus*, *Pseudomonas*, *Corynebacterium*, *Arthrobacter*, and *Micrococcus* were found to be capable of PUR biodegradation [71,72,73]. Again, a wide range of esterases have been identified as active enzymes involved in depolymerization, including lipases, cutinases, and proteases.

Even though some organisms have been shown to act on plastics with a C-C backbone, the extent of specific enzymes involved in the direct or indirect cleavage of these polymers is still scarce [67,74]. In fact, the plastics-active enzyme database (PAZy) only lists two reported enzymes unambiguously involved in the oxidation of PE, while no enzymes have been described so far for the degradation of PP, PVC, and PS [75]. It is assumed that the short presence of C-C polymers in the environment was not sufficient enough for a strong evolutionary adaption to these materials as substrates [76]. Still, some microorganisms capable of growing on PE, PP, PVC, or PS have been reported by several authors [66]. In the 1990s, Lee et al. [77] described the decrease of the molecular weight (MW) of PE samples after incubation with certain species of *Streptomyces* spp., which are known as natural lignin-degrading organisms. The ability of different fungi such as *Aspergillus* spp., *Pseudomonas* spp., and *Bacillus* spp. to utilize low-density polyethylene (LDPE) as a carbon source was elucidated by Zahra et al. [78] and Muhonja et al. [79].

Furthermore, findings from Iiyoshi et al. [80] and Zhao et al. [81] indicated a reduction of the MW and mechanical properties of PE by the incubation with Mn-peroxidase and soybean peroxidase, respectively. The oxidation of the plastic utilizing H_2_O_2_ as an oxidizing agent supposedly led to the formation of surface radicals by the abstraction of hydrogen from the polymer. This is expected to have led to the introduction of hydroxyl groups on the surface by molecular oxygen [81]. Recently, Sanluis-Verdes et al. [82] published the only two enzymes also recognized by PAZy in the context of oxidative degradation of PE. They identified an arylphorin and hexamerin in the saliva of *Galleria mellonella* larvae involved in the unspecific oxidation of PE films and powder. In addition to the increase of the carbonyl peak in Raman and FTIR (Fourier-transform infrared spectroscopy) spectra, 2-ketones and additives could be detected by mass spectrometry after treatment with the larvae saliva. Thus, until today, this is the only comprehensively described study in which the enzymes involved in the degradation of C-C polymers have been identified.

### 3.2. Approaches towards Epoxy Biodegradation

In the case of epoxy resins and epoxy-based fiber-reinforced polymers, different approaches towards the biochemical degradation have been taken (see Table 3). Eliaz et al. [83] investigated the growth of microbes from soil samples from an epoxy and PUR manufacturing site in Israel in minimal medium containing droplets of two commercially available epoxy resins (Araldite^®^ LY 5052, Huntsman Corp., The Woodlands, TX, USA; EPON^™^ 815C, Hexion, Columbus, OH, USA). They identified the bacteria *Rhodococcus rhodochrous* and *Ochrobactrum anthropi* to be able to grow on the BPA-based LY 5052 resin by consecutive enrichment cultures performed over the course of five weeks. Interestingly, a synergistic effect was observed, since both organisms were unable to utilize the resin on their own. The authors stated the possibility of the joint action of two or more enzymes, which only enables the complete degradation of the resin when the other species is present. However, the involved enzymes are yet to be elucidated, as well as the synergy between both organisms.

The effect of the presence of *Pseudomonas putida* on the corrosion resistance of marine epoxy resin varnish coatings was investigated by Wang et al. [84]. Epoxy-coated (BPA-based) steel samples were incubated with the bacterium in seawater and sterile seawater was used as a negative control. The decrease in corrosion resistance was significantly higher for the samples containing *P. putida* and a strong biofilm formation was observed, which indicated the ability of the organism to grow on the epoxy coating. Fourier-transform infrared spectroscopy (FTIR) measurements revealed an intensity decrease of the bands associated with the hydroxy groups (C-OH), leading to the speculation of the oxidation of these groups to the corresponding carbonyls.

Another marine microorganism known to be able to accelerate the corrosion of metals, *Pseudomonas aeruginosa* (1A00099), was chosen by Zhang et al. [85] for incubation with epoxy coatings (BADGE hardened with Jeffamine D230) on carbon steel. The authors conducted immersion tests of the epoxy coatings in sterile and inoculated culture media with different nutrient concentrations (100%, 10%, and 0%) for 28 days. They used various techniques to evaluate the coating degradation and corrosion behavior, such as electrochemical impedance spectroscopy (EIS), FTIR, scanning electron microscopy (SEM), and energy dispersive spectroscopy (EDS). They found that *P. aeruginosa* promoted the deterioration of epoxy coating by destroying the C-O-C and C-O groups, which are important for the cross-linking and adhesion of the coating. The authors also concluded that the coating suffered more damage under starvation conditions as the number of *P. aeruginosa* cells attached to the coating surface increased, and the biofilm became denser and more complex.

Three different *P. aeruginosa* strains (MTCC 7815, MTCC 7814, and PN8A1) were investigated by Dutta et al. [86] in the context of biodegradation of epoxy and melamine formaldehyde (MF)-modified PU films from the oil of *Mesua ferrea* seeds (MFLSO). Polyurethane resins were prepared from MFLSO and blended with epoxy and MF resins in various proportions. The biodegradation of the films was evaluated by two different methods: targeted microbial degradation using cultures of the three *P. aeruginosa* strains and natural soil burial degradation under ambient conditions. Bacterial growth in the polymer matrix, weight loss and mechanical properties of the films and chemical changes by FTIR spectroscopy were measured. The authors found that both epoxy- and MF-modified PU films were biodegradable to some extent, but epoxy-modified films showed higher biodegradation rates than MF-modified films. They attributed this to the higher hydrophilicity and lower cross-linking density of epoxy-modified films, which was suggested to facilitate the penetration and attack of microorganisms.

**Table 3:** Reported strains or isolated enzymes in the context of epoxy biodegradation.

Tested Resin	Applied Biocatalyst(s)	Analytics	Ref.
Araldite® LY 5052, Huntsman Corp., The Woodlands, TX, USA EPON™ 815C, Hexion, Columbus, OH, USA	*Rhodococcus rhodochrous* *Ochrobactrum anthropi*	Turbidity measurement	[83]
BPA-based epoxy, (not specified)	*Pseudomonas putida*	EIS, SEM, FTIR, Contact angle measurement	[84]
Epoxy varnish, (not specified)	*Bacillus flexus*	EIS, SEM, FTIR	[87]
Epoxy and epoxy-silicone blends, (not specified)	*Bacterium Te68R* *Microbacterium* sp. (strain MK3) *Pseudomonas putida*	FTIR, TGA	[88]
Epoxy resin L + hardener GL21, Suter Kunststoffe, Fraubrunnen, Bern, Switzerland	*Ganoderma adspersum*	Weight loss, Contact angle measurement, Mechanical testing, FTIR	[89]
Epoxy Hercules 3501-6	*Aspergillus versicolor* *Cladosporium cladosporioides*	EIS, SEM, Mechanical testing	[90]
Carbon-fiber-reinforced epoxy and glass- and carbon-fiber-reinforced vinyl ester (not specified)	*Thiobacillus ferroxidans* *Pseudomonas fluorescens* *Lactococcus lactis* *Clostridium acetobutylicum*	SEM, Mechanical testing	[91]
Hexflow^®^ RTM-6 model compound *N*,*N*-bis(2-hydroxypropyl)-p-toluidine (NNBT)	Unspecific peroxygenases *Agrocybe aegerita*—PaDa-I *Psathyrella aberdarensis*—GroGu *Marasmius rotula*—rMroUPO *Psathyrella aberdarensis*—PabUPOII	GC-MS	[92]
PU blend with BPA-based epoxy, amine-hardened (not specified)	*Pseudomonas aeruginosa* (MTCC 7815, MTCC 7814, PN8A1	Weight loss, FTIR, TGA, SEM, Mechanical testing	[86]
BADGE hardened with Jeffamine D230	*Pseudomonas aeruginosa* (1A00099)	Contact angle measurement, Mechanical testing, FTIR, SEM	[85]

Deng et al. [87] conducted a similar study in which coupons, coated with an epoxy varnish, were immersed in sterile seawater containing *Bacillus flexus* for up to 30 days. EIS revealed a strong decrease in corrosion resistance during the first 19 days of incubation, which was interpreted to be a direct result of the degradation of the varnish coating by the microorganism. Further evidence of an interaction of *B. flexus* with the coating was given by electron microscopy, revealing the formation of a bacterial biofilm on the varnish. The presence of depositions and holes in the surface led the authors to speculate about the influence of secreted organic acids, which are assumed to have accelerated the degradation of the coating.

The use of two different bacterial consortia for the biodegradation of cured epoxy samples was tested by Negi et al. [88]. The consortium containing *Bacterium Te68R*, *Microbacterium* sp. (strain MK3), and *P. putida* exhibited the maximum growth in minimal medium containing crushed epoxy in a concentration of 5 g∙L^−1^ as C-source, leading to a weight loss of 34.17% over the course of 15 days. The authors used FTIR spectroscopy to trace the alterations in the chemistry of the epoxy and found a reduction of the peaks associated with BPA. Hence, a partial breakdown of the polymeric backbone was hypothesized as a result of the microbial attack.

Exploiting the chemical similarities of lignin to cured epoxy, Pardi-Comensoli et al. [89] approached the epoxy degradation by utilizing *Ganoderma adspersum*, a fungus known to produce lignin peroxidase and laccase. As these two enzymes are predominantly involved in lignin degradation, it was also assumed, that they would act on epoxy. The authors used plates of epoxy L, a BPA/BPF (Bisphenol F)-based resin, which was cured with the aromatic amine hardener GL21 (both from Suter Kunststoffe, Fraubrunnen, Bern, Switzerland) for incubation experiments in sterile and non-sterile soil with different water holding capacities (60 and 90%, respectively) with the fungus to resemble the natural microcosm of the organism. Analysis of the plates was conducted after 1, 3, and 6 months by weight loss, comparison of hardness and flexure, as well as contact angle measurements and FTIR. However, after incubation, no clear tendency concerning the weight loss of the samples could be determined and the hardness and flexure of the material did not change significantly. Only a reduction of the hydrophobicity was detected by the contact angle measurement, which indicated an oxidation of the surface. Therefore, no unambiguous conclusion about the biodegradation with *G. adspersum* could be drawn.

The first attempts concerning the microbial degradation of fiber-reinforced composite materials were pursued in the 1990s. Gu et al. [90] used a fungal consortium of *Aspergillus versicolor*, *Cladosporium cladosporioides*, and *Chaetomium* sp. for degradation experiments with a graphite–fiber-reinforced composite of the Hercules 3501-6 epoxy resin. The cured material was incubated with the consortium in malt broth medium in shake flasks. After an incubation time of 30 days, the colonization and penetration of the material by the fungi and their hyphae was confirmed by SEM. Further characterization was conducted by EIS, which revealed an increase in the pore sizes and number. By testing the mechanical properties, a decrease in the bonding strength between the matrix and fibers was observed after incubation. Additionally, aqueous extracts of the cured resins (prepared by autoclaving) were used in minimal medium to examine the effect on fungal growth. The authors stated that cell proliferation was significantly promoted but did not further investigate the composition of the extracts.

A different approach was taken by Wagner et al. [91], who tested different bacterial strains aiming at specific degradation mechanisms. They employed *Thiobacillus ferroxidans* (sulfur/iron-oxidizing bacterium), *Pseudomonas fluorescens* (calcareous-depositing bacterium), *Lactococcus lactis* (ammonium-producing bacterium), *Clostridium acetobutylicum* (hydrogen-producing bacterium), and a mixed facultative/anaerobic marine culture of sulfur-reducing bacteria for degradation experiments with neat epoxy and composites with carbon-fibers. Even though all of the strains colonized the surface of the test samples, no degradation and/or decrease of mechanical properties could be observed.

The first systematical investigation of the application of isolated enzymes in the context of epoxy degradation was published by Dolz et al. [92]. The authors developed a colorimetric screening assay aimed at engineering unspecific peroxygenases (UPOs) for the degradation of epoxy resins, using Hexflow^®^ RTM-6, a commercial epoxy resin applied extensively in the aeronautics sector. They used mutants from the short (*Marasmius rotula*) and long (*Agrocybe aegerita*, *Psathyrella aberdarensis*) UPO family to determine their potential *N*-dealkylation activity on *N*,*N*-bis(2-hydroxypropyl)-p-toluidine (NNBT) as a main structural scaffold of Hexflow^®^ RTM-6 and established a directed evolution platform to engineer composite degrading variants. However, experimental approaches towards the targeted degradation of the resin itself are still pending.

## 4. Analytical Methods for Characterization of Bio-Degraded Epoxy

**Figure 7:**
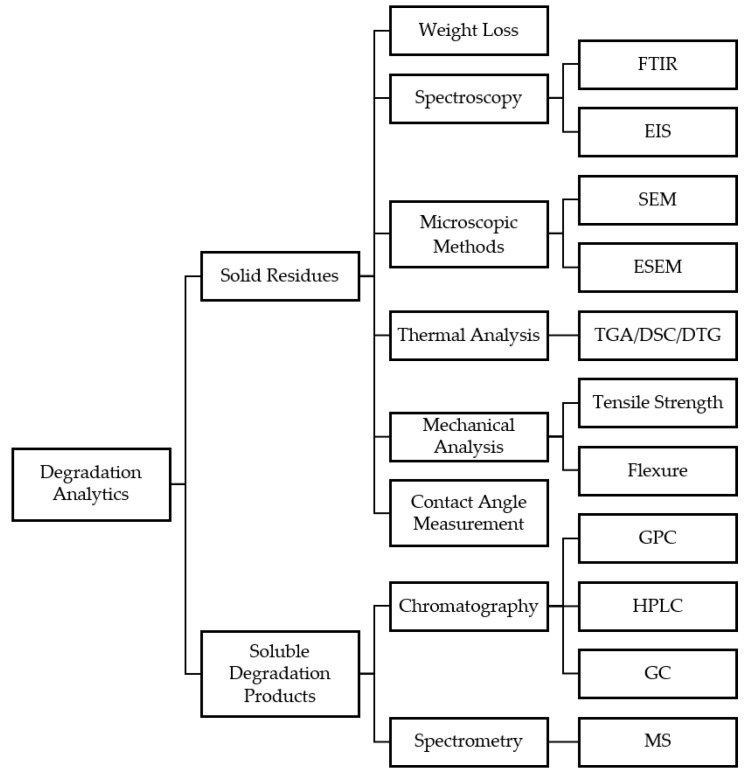
Overview on analytical methods for the analysis of epoxy biodegradation; FTIR—Fourier-Transform Infrared Spectroscopy, EIS—Electrochemical Impedance Spectroscopy, SEM—Scanning Electron Microscopy, ESEM—Environmental Scanning Electron Microscopy, TGA—Thermogravimetric Analysis, DSC—Differential Scanning Calorimetry, DTG—Differential Thermogravimetry, GPC—Gel Permeation Chromatography, HPLC—High Performance Liquid Chromatography, GC-—Gas Chromatography; MS—Mass Spectrometry.

The analysis of the biodegradation of plastics is of vital importance for the characterization and assessment of the involved biocatalysts. Several methods have been discussed in the literature so far to qualitatively and quantitatively monitor the depolymerization of plastics. Different authors have pointed out the need for reliable and robust analytical methods, that deliver irrevocable evidence for the degradation of the plastic [65,93,94]. In this respect, the solid residues of the treated sample and the soluble degradation products can be analyzed to allow a comprehensive study of the mechanisms involved (see Figure 7). Therefore, in the following section, analytical methods applied to investigate epoxy degradation will be covered.

### 4.1. Weight Loss

Weight loss measurement is a commonly used analytical tool to evaluate enzymatic or microbial plastic degradation. The method involves measuring the loss of mass of a sample of plastic material after exposure to a biocatalyst. This weight loss is an indication of the extent of degradation that has occurred. Weight loss measurement is a simple and cost-effective technique, and it can be used to determine the rate and degree of plastic degradation in different environments [95]. However, the plain weight comparison of the untreated and degraded sample was criticized as being too vague to judge the backbone depolymerization of plastics, since this measurement method is heavily influenced by systematic errors arising from the presence of additives such as plasticizers, that might be broken down instead of the polymer itself [67]. Since this method also requires a measurable change in the sample weight and can be difficult to implement if, for example, ground samples are used to increase the surface area, it is only suitable to a limited extent and should be used in conjunction with other analytical techniques to ensure accurate and reliable results [96].

### 4.2. Microscopic Methods

Microscopic methods offer a fast and reliable way to analyze sample surfaces and are therefore excellently suited for an initial qualitative assessment [97]. In this respect, scanning electron microscopes (SEM) offer the highest performance, reaching spatial resolutions down to 1 nm, whereby even subtle changes in the morphology can be detected. In contrast to light microscopes, whose resolution depends on the wavelength of light in the visible spectrum (≥200 nm), electron microscopes utilize an accelerated focused electron beam with a 55,000-fold shorter wavelength (≥0.0037 nm) [98]. After striking a spot on the sample surface, the primary electron beam transfers a certain kinetic energy to the atoms of the material, causing secondary electrons to be released which provide information about the structure of the sample. The beam captures the surface along a grid, enabling a highly detailed image of the surface [99]. To avoid any interference of the electrons with the gases in the atmosphere, SEM analysis was carried out in a high vacuum, eliminating the possibility of measuring liquid samples. However, a modified SEM method called environmental scanning electron microscopy (ESEM) has been developed, which permits a certain pressure in the sample compartment through a two-chamber system, allowing the measurement of liquids or volatile samples [100].

Electron microscopy was used by several authors for the observation of structural changes of the sample surface and for the elucidation of microorganism morphology in the context of epoxy biodegradation. SEM micrographs allowed Gu et al. [90] to observe the fungal colonization of carbon-fiber-reinforced polymer samples, which revealed a penetration of the fungal hyphae into the composite and surface fractures. Pangallo et al. [101] qualitatively demonstrated the growth of *Rhizopus microsporus* and *Aspergillus fumigatus* on epoxy samples by visual inspection of the surfaces. The SEM analysis revealed a deposition of mineral crystals on the hyphae of *R. microsporus*, which were suspected to be resulting from the mineralization of the material. Apart from cured samples, ESEM was used for the analysis of uncured epoxy resin droplets originating from incubation experiments with microbial consortia by Eliaz et al. [83]. The authors were able to visualize the increase of surface roughness of the resin droplets as a result of microbial attack. ESEM was also employed in experiments from Pardi-Comensoli et al. [89], who screened cured epoxy samples for attached microorganisms after incubation in microbiological water. The morphological inspection of the surfaces facilitated the identification of the types of microorganisms interacting with the epoxy and revealed similar crystal depositions as described earlier. Microscopic analysis can therefore be considered as a method for initial qualitative inspection of plastic samples, which harbors the potential to elucidate not only alterations in the surface but also give hints on the metabolization of the material by biocatalysts.

### 4.3. Spectroscopic Methods

Attenuated total reflection Fourier-transform infrared spectroscopy (ATR-FTIR) is widely used in the analysis of plastics and their degradation due to its rather simple setup and good reproducibility of the measurements [102]. The technique relies on the vibrational excitation of chemical bonds by infrared radiation, leading to the absorption of certain frequencies corresponding to the chemical structure of the analyte. Given the typical repetitive nature of polymers, FTIR spectroscopy usually reveals the chemical structure of the involved monomers and bonds between them. The so-called fingerprint region (approximately 1400–400 cm^−1^) of the resulting spectra contains a large part of the bands specific to the respective polymer, which are caused by fundamental stretching vibrations, couplings of these, and deformation vibrations [103].

The method was applied, for example, in the study of microbial degradation of vinyl ester based carbon-fiber-reinforced composites by Breister et al. [19]. They observed the increase in the intensity of peaks associated with the -O-H, -C-H, and -C=O stretching, and assigned these changes to the scission of ester bonds in the matrix polymer. Zahra et al. [78] employed FTIR spectroscopy to trace the photo-oxidative pretreatment of PE films by ultraviolet (UV) radiation and the subsequent degradation of the material by fungal attack. They were able to identify a band at 1720 cm^−1^ related to the -C=O stretching vibration introduced by the UV irradiation. In further experiments, a subsequent decrease of the peak was observed as a result of the incubation with different fungi, leading to the assumption of microbial degradation of the PE films. An even more comprehensive analysis is made possible by FTIR microscopes, which allow, for example, the investigation of the distribution of functional groups on the surface of samples. Eibl [104] used such an instrument to examine the thermal degradation of an epoxy-based CFRP. Vertical sliced samples of the heat-treated composite were monitored under the FTIR microscope, which revealed a decreased thermal decomposition towards the inner layers of the material. This enables a combination of microscopic analysis and structural elucidation of the respective surfaces.

Another commonly applied spectroscopic methods is electrochemical impedance spectroscopy (EIS), which involves measuring the electrical response of a plastic material in a particular environment, allowing for the determination of changes in the material’s properties over time. EIS is particularly useful for evaluating the degradation of plastics in aqueous environments, where the electrical properties of the material can be influenced by the presence of microorganisms and enzymes. The method is non-destructive and can be performed in situ, making it an attractive technique for monitoring the progress of plastic degradation over time [105]. In EIS, a small alternating current was applied to the analyte, and the resulting current response was measured. This current response provides information on the electrical properties of the plastic material, including its resistance, capacitance, and frequency-dependent behavior [106]. Changes in these electrical properties can be indicative of plastic degradation. For example, Wang et al. [84] correlated the presence of microorganisms and the concurrent degradation of the surface of epoxy coated steel coupons to a decrease in resistance and an increase in capacitance.

Zhang et al. [85] applied EIS and measured the potentiodynamic polarization of epoxy coatings to evaluate their degradation by *P. aeruginosa*. They found that the presence of the bacteria promoted the degradation of the epoxy coating, especially under starvation conditions, and that the coating had lower low frequency impedance modulus and higher corrosion current density in the inoculated medium with starvation conditions. Additionally, a notable rise in the phase angle of the coating was observed, which serves as an indicator of an increased water absorption and concomitant reduction in barrier properties of the epoxy.

### 4.4. Thermal Analysis

Thermogravimetric analysis (TGA) is a measurement method in which a sample is constantly heated at a fixed rate to monitor the relationship between the mass and the temperature—usually under a controlled atmosphere such as nitrogen, argon, or oxygen. The resulting TGA curve and its first derivative, referred to as differential thermogravimetric (DTG) curve, reveal valuable information for the characterization of the analyte. Typically, a step-wise decrease in mass can be observed, which might be associated, e.g., with the evaporation of volatile compounds or thermal degradation and is therefore specific for a certain substance or material [107]. Therefore, in polymer analysis, material properties such as the thermal stability, degree of curing, or even composition can be derived from the data. This is usually reflected in a decrease in the thermal onset degradation temperature and a generally higher weight loss [108].

The application of simultaneous differential thermal analysis for the evaluation of biochemical plastic degradation was described by Negi et al. [88]. They analyzed the thermal behavior of a control and degraded epoxy sample from 20 to 550 °C under an inert nitrogen atmosphere at a heating rate of 5 °C∙min^−1^ and compared the TGA and DTG curves. While thermal degradation of the untreated epoxy started at 325 °C and stopped at 425 °C with a weight loss of 19.05%, it occurred already at lower temperatures for the bacteria-incubated samples and showed higher losses of up to 34.17%.

Another example was published by Breister et al. [19], who also utilized TGA for the characterization of carbon-fiber-reinforced vinyl ester composites that were incubated with a microbial consortium. The measurements allowed for tracing the ongoing decrease in the thermal degradation onset temperature over a course of several weeks as an indicator for the microbial degradation. Since it can generally be assumed that thermal degradation occurs at lower temperatures due to the reduced molecular weight of degraded samples, thermal analysis is well suited for estimating the effectiveness of biocatalysts involved in the depolymerization.

### 4.5. Chromatographic Methods

While surface analytical and thermal methods do not necessarily allow for a direct analysis of the products of a degraded polymer, chromatographic methods make it possible to specifically investigate the individually formed oligo- and monomers. This opens further possibilities to trace the degradation process by monitoring the changes in the molecular weight distribution of a polymer or to elucidate the involved mechanisms by separation and identification of the resulting degradation products. The size of the fragments resulting from the degradation is decisive and determines which separation technique is used. For larger polymer fragments of a molecular weight (MW) > 2000 Da, Mestan and Morris [109] proposed the use of gel permeation chromatography (GPC), whereas smaller molecules can also be separated by means of other separation mechanisms such as adsorption, ion exchange, or hydrophobic interaction.

An example for the utilization of GPC for the measurement of changes in the molecular weight distribution was given by Yan et al. [110] for an epoxy resin after treatment with supercritical 1-propanol. The chromatograms were recorded with an UltiMate 3000 liquid chromatography system (Thermo Fisher Scientific, Waltham, MA, USA) employing three Styragel columns (Waters, Milford, MA, USA). The calibration was carried out with polystyrene standards, which allowed the peaks to be assigned to the corresponding molecular weights. The chromatograms showed that the initially relatively narrow distribution of the components of the resin was broadened by the physicochemical treatment, leading to the assumption that degradation of the cured epoxy took place.

The resolution of lower molecular weight distributions was demonstrated by Fuchslueger et al. [111]. They characterized a commercially available BPA-based epoxy resins concerning its individual constituents and by-products by reverse-phase (RP) chromatography, employing an Ultremex C18-column (Phenomenex, Torrance, CA, USA) with an acetonitrile (ACN) gradient. The detection of the fractions was conducted with an UV-detector followed by a quadrupole mass spectrometer, allowing for the assignment of molecular weights. With this, a complete set of mono and oligomers was derived from the data ranging from around to 350 to 2300 Da, which resembled a separation of oligomers up to 24 repeating units.

The analysis of single components of the epoxy matrix of a partially cured fiber-reinforced composite by RP-chromatography was described by Noël et al. [112]. They extracted the epoxy components from the composite with tetrahydrofuran (THF) and separated them on a C8-column (Hypersil MOS-1; Thermo Fisher Scientific, Waltham, MA, USA), utilizing a linear ACN gradient. By comparison to reference spectra of the monomeric compounds, the main resin ingredients such as 4,4′-diaminodiphenyl sulfone (DDS), 4,4′-methylenbis[*N*,*N*-bis(2,3-epoxypropyl)anilin] (TGDDM) and DGEBA were identified. The quantification of the compounds was realized using acetophenone as an internal standard to enable a comparison of the mass fractions of different batches of the resin.

In addition to liquid chromatography, gas chromatography with coupled mass spectrometry (GC-MS) was used for the analysis of solvolyzed CFRPs [113]. The authors prepared cured composites with a DGEBA/DDS-based epoxy matrix and subjected them to different hydrolysis conditions and organic solvents to facilitate the solvolysis of the polymer. A HP-5MS GC-column (Agilent, Santa Clara, CA, USA) was used for the separation of the degradation products, which were detected with a time-of-flight (TOF) MS. Up to 15 different degradation products were identified from the mass spectra by a survey of the NIST MS spectra library, revealing a variety of aromatic species such as aniline, quinoline, phenol, and respective (amine-)derivatives.

### 4.6. Contact Angle Measurement

The contact angle is the angle formed by a liquid drop at the interface with a solid surface. It reflects the surface wettability of the solid, which is related to its surface energy and chemical composition [114]. A high contact angle indicates a low surface energy and a hydrophobic surface, while a low contact angle indicates a high surface energy and a hydrophilic surface. Contact angle measurement can be performed by sessile-drop goniometry, which involves recording a video of a water drop on a plastic surface and determining the contact angle by image analysis. By comparing the contact angles of pristine and degraded plastics, the extent of degradation and the changes in surface properties can be assessed. A decrease in contact angle suggests that the plastic surface has become more polar and oxidized due to degradation, while an increase in contact angle suggests that the plastic surface has become more non-polar and rough due to degradation [115].

The application of contact angle measurement for epoxy degradation analysis was conducted by Pardi-Comensoli et al. [89]. They used a goniometer to measure the contact angle of water droplets on the epoxy resin samples before and after soil incubation. The results showed that the contact angle of the epoxy resin samples decreased significantly after microbial exposure, indicating that the microbes can reduce the hydrophobicity of the epoxy resin surface. The authors suggested that this reduction in hydrophobicity could facilitate the ingress of water into the epoxy resin matrix and enhance the microbial depolymerization process.

A similar approach was followed by Zhang et al. [85], who analyzed the increasing hydrophilicity of epoxy coatings on steel specimen over time by measurement of the water contact angle They observed a reduction in the contact angle, from 70° to 63°, upon incubation of the coated samples with *P. aeruginosa* for 14 days. This decrease in contact angle suggests the formation of a biofilm on the surface, as reported by the authors.

### 4.7. Mechanical Analysis

Mechanical analysis involves measuring the mechanical properties of plastics, such as tensile strength, elongation, modulus, impact resistance, and hardness. These properties reflect the molecular structure and chain length of plastics, which can be altered by degradation processes. Mechanical testing can be performed by using standard instruments and methods, such as universal testing machines, impact testers, hardness testers, and dynamic mechanical analysis [95].

The mechanical properties of epoxy- and melamine formaldehyde-modified PU films were measured before and after soil burial biodegradation for 180 days by Dutta et al. [86]. The tensile strength and elongation at break of the films were determined by a universal testing machine. The results showed that the polyurethane/epoxy blends had higher tensile strength and elongation at break than the polyurethane/MF blends, and both types of blends showed a significant loss of mechanical properties after biodegradation.

## 5. Challenges and Opportunities

Epoxy resins have gained tremendous importance in the industrial sector as they possess superior mechanical and thermal properties, making them an ideal choice for various applications [34,58]. The increasing demand for epoxy resins is expected to continue in the future, especially in the production of fiber-reinforced composites [116]. However, conventional methods for epoxy recycling are energy-intensive and use toxic chemicals, highlighting the need for sustainable alternatives such as biological methods [117].

While biological plastic recycling has mostly focused on thermoplastics, research on more recalcitrant plastics such as epoxy is limited [75]. There have been a few studies suggesting that some microorganisms might be capable of degrading epoxy, but unambiguous proof about the involved enzymes is still not available. In this regard, the high durability and cross-linked structure of epoxy resins are significant hurdles in the biochemical degradation process, as the materials are insoluble in aqueous reaction systems, reducing accessibility for biocatalysts [118]. Moreover, their hydrophobic nature and high molecular weight further limits the attachment of microbial biofilms and might hinder the targeted action of an enzyme [83,119].

**Figure 8:**
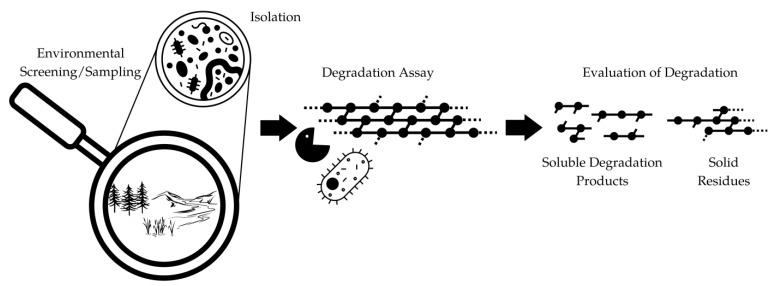
Overview on the biocatalyst identification and testing workflow based on environmental sampling and/or metagenomic screening.

To overcome these challenges, novel approaches need to be developed to extend the range of biodegradable polymers. One potential solution is to exploit the natural biodiversity, especially microorganisms that have not yet been cultivated or cannot be cultivated at all [24,120]. The so-called microbial dark matter likely harbors promising biocatalysts accessible through metagenomic approaches (see Figure 8) [121]. Additionally, state-of-the-art molecular biology methods such as protein engineering can be used to create new or improved biocatalysts that might be able to act on epoxy.

Furthermore, the targeted use of oxidative enzymes has emerged as a promising strategy for the degradation of non-hydrolyzable polymers. By studying reaction conditions and enzyme formulations, researchers have made progress in enhancing the degradation efficiency of recalcitrant plastics such as PE using oxidative enzymes [122,123]. These enzymes, such as peroxidases and laccases, possess the ability to initiate and propagate radical reactions, enabling the breakdown of complex structures, and might be applicable to epoxy as well [74]. However, further studies are needed to overcome challenges such as limited enzyme stability and substrate accessibility, thereby unlocking the full potential of oxidative enzymes for epoxy recycling.

Apart from recycling approaches for conventional, fossil-based epoxy, research on bio-degradable epoxy and bio-reinforced resins is also of high importance. Novel bio-degradable epoxy resins have been developed, such as those based on soy protein and lignin [124,125]. These materials offer good mechanical properties, thermal stability, and biodegradability, making them a viable alternative to conventional epoxy resins [126]. Bio-reinforced resins, on the other hand, use natural fibers or fillers to enhance the mechanical properties of the epoxy matrix. This approach can lead to improved properties while reducing the amount of synthetic resin required, thus reducing the environmental impact [10].

In conclusion, the recycling of epoxy resins with bio-based methods is an important area of research, as it offers a sustainable and environmentally friendly alternative to conventional methods. While challenges exist in terms of recalcitrant epoxy degradation and the need for robust biocatalysts, advancements in metagenome mining, directed evolution, and utilization of oxidative enzymes provide avenues for overcoming these hurdles. Additionally, research on bio-degradable epoxy and bio-reinforced materials contributes to a holistic approach towards a more sustainable epoxy industry. Continued interdisciplinary efforts in exploring and optimizing bio-based epoxy recycling methods will pave the way for a greener future and a circular economy.

## Figures and Tables

**Table 1:** Mechanical properties of epoxy, carbon-fiber-reinforced polymers (epoxy-based), and stainless steel.

Property	Unit	Epoxy	CFRP	Stainless Steel
Density	g∙cm^−3^	1.1–1.4	1.5–2.1	7.85
Tensile Strength	MPa	60	600–3920	483–690
Young’s Modulus	GPa	2.5	37–784	200
Elongation	%	-	0.5–1.8	6–12
Coefficient of Linear Expansion	10^−6^∙°C^−1^	-	−9–0	11.7
Reference		[48]	[49]	

**Table 2:** Overview of the hetero- and homoatomic backbone classification of selected synthetic polymers (adapted from [43]).

	Name	Structure
**heteroatomic backbone**	Polyethylene terephthalate (PET)	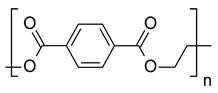
	Polyamide (PA)	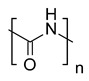
	Polylactic acid (PLA)	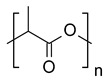
	Epoxy	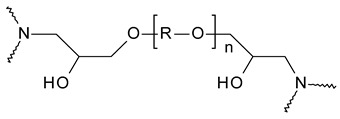
	Polyurethanes (PU)	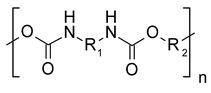
**homoatomic backbone**	Polyethylene (PE)	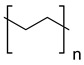
	Polypropylene (PP)	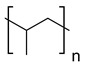
	Polyacrylamide (PAM)	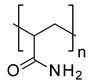
	Polyvinyl chloride (PVC)	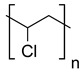
	Polystyrene (PS)	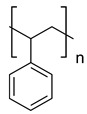

## Data Availability

The data presented in this study are available on request from the corresponding author.
